# Endocannabinoids and related *N*-acylethanolamines: biological activities and metabolism

**DOI:** 10.1186/s41232-018-0086-5

**Published:** 2018-10-01

**Authors:** Kazuhito Tsuboi, Toru Uyama, Yasuo Okamoto, Natsuo Ueda

**Affiliations:** 1 Department of Biochemistry, Kagawa University School of Medicine, 1750-1 Ikenobe, Miki, Kagawa 761-0793 Japan; 2 Department of Pharmacology, Kawasaki Medical School, 577 Matsushima, Kurashiki, Okayama 701-0192 Japan

**Keywords:** Lipid mediator, Endocannabinoid, 2-Arachidonoylglycerol, Anandamide, *N*-Acylethanolamine, Metabolism, Phospholipid, Phospholipase

## Abstract

The plant *Cannabis sativa* contains cannabinoids represented by Δ^9^-tetrahydrocannabinol, which exert psychoactivity and immunomodulation through cannabinoid CB1 and CB2 receptors, respectively, in animal tissues. Arachidonoylethanolamide (also referred to as anandamide) and 2-arachidonoylglycerol (2-AG) are well known as two major endogenous agonists of these receptors (termed “endocannabinoids”) and show various cannabimimetic bioactivities. However, only 2-AG is a full agonist for CB1 and CB2 and mediates retrograde signals at the synapse, strongly suggesting that 2-AG is physiologically more important than anandamide. The metabolic pathways of these two endocannabinoids are completely different. 2-AG is mostly produced from inositol phospholipids via diacylglycerol by phospholipase C and diacylglycerol lipase and then degraded by monoacylglycerol lipase. On the other hand, anandamide is concomitantly produced with larger amounts of other *N*-acylethanolamines via *N*-acyl-phosphatidylethanolamines (NAPEs). Although this pathway consists of calcium-dependent *N*-acyltransferase and NAPE-hydrolyzing phospholipase D, recent studies revealed the involvement of several new enzymes. Quantitatively major *N*-acylethanolamines include palmitoylethanolamide and oleoylethanolamide, which do not bind to cannabinoid receptors but exert anti-inflammatory, analgesic, and anorexic effects through receptors such as peroxisome proliferator-activated receptor α. The biosynthesis of these non-endocannabinoid *N*-acylethanolamines rather than anandamide may be the primary significance of this pathway. Here, we provide an overview of the biological activities and metabolisms of endocannabinoids (2-AG and anandamide) and non-endocannabinoid *N*-acylethanolamines.

## Background

**Fig. 1:**
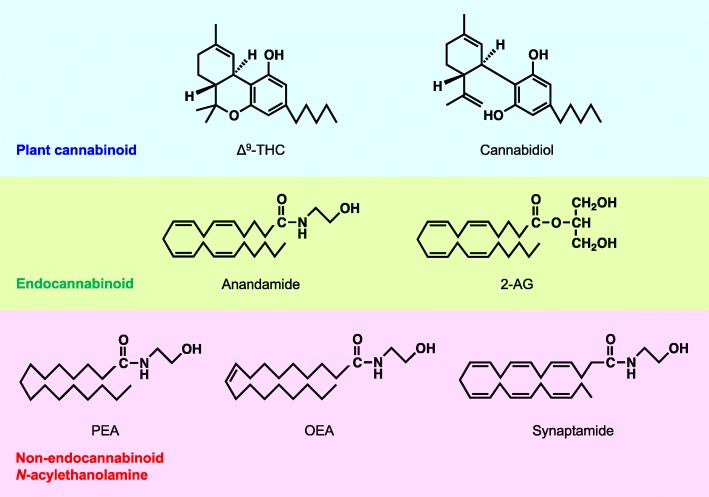
Chemical structures of representative plant cannabinoids, endocannabinoids, and non-endocannabinoid *N*-acylethanolamines

Preparations of the plant *Cannabis sativa*, such as marijuana and hashish, have been used for recreational and medical purposes for thousands of years [1]. The oldest written description of medicinal cannabis dates back to around 2350 B.C., which was found on a stone from the pyramids in Egypt. Although their psychoactivities, including euphoria, hallucination, and analgesia, have been known for a long time, the purification of Δ^9^-tetrahydrocannabinol (Δ^9^-THC) as the major psychoactive constituent, followed by the determination of its chemical structure, was not achieved until the 1960s [2] (Fig. 1). A large number of structurally related compounds were also isolated from cannabis and collectively referred to as cannabinoids. Synthetic analogs with more potent cannabimimetic activities were also developed and used to pharmacologically characterize a specific receptor for cannabinoids existing in rat brain crude membrane preparations [3]. The central-type CB1 cannabinoid receptor was then molecularly identified by its cDNA cloning in 1990 [4]. Subsequently, cDNA of the peripheral-type CB2 cannabinoid receptor was also found by using its sequence similarity to CB1 receptor [5]. In contrast to Δ^9^-THC, cannabidiol, another major cannabinoid in cannabis, showing anti-inflammatory and anticonvulsive effects, was almost inactive for cannabinoid receptors. Since cannabinoids are derived from the plant cannabis but not from mammals, animal tissues were expected to have endogenous counterparts capable of binding to cannabinoid receptors (later termed “endocannabinoids”). Arachidonoylethanolamide, the ethanolamide of arachidonic acid, was isolated as the first endocannabinoid from pig brain and named anandamide after “ananda,” which means bliss in Sanskrit [6] (Fig. 1). Shortly after that, another derivative of arachidonic acid, 2-arachidonoylglycerol (2-AG), was also reported to show the same agonistic activity [7, 8]. It was surprising since 2-AG has been known for a long time simply as a common intermediate in the metabolisms of glycerophospholipids and triglyceride. Currently, 2-AG and anandamide are considered to be a full agonist and a partial agonist of cannabinoid receptors, respectively. Arachidonic acid is a polyunsaturated fatty acid (20:4) well known as the precursor of bioactive prostaglandins and other eicosanoids. Endocannabinoids are thus considered to be other members of arachidonic acid-related lipid mediators.

In addition to anandamide, ethanolamides of various long-chain fatty acids are also present in the body. These ethanolamides, including anandamide, are collectively referred to as *N*-acylethanolamines (Fig. 1). Ethanol-amides of saturated and monounsaturated fatty acids such as palmitic (16:0), stearic (18:0), and oleic acids (18:1) are much more abundant than anandamide in the body. These saturated and monounsaturated *N*-acylethanol-amines do not bind to cannabinoid receptors, but they can activate peroxisome proliferator-activated receptor α (PPARα), a nuclear receptor, and other receptors, leading to the exertion of biological activities including anti-inflammation and appetite suppression. In this mini-review, we will outline the biological activities and metabolisms of endocannabinoids and related *N*-acylethanolamines and emphasize that 2-AG is physiologically more important than anandamide, which appears to be a minor component concomitantly produced with cannabinoid receptor-insensitive *N*-acylethanolamines.

## Biological activities of endocannabinoids

CB1 and CB2 cannabinoid receptors are G protein-coupled receptors possessing seven transmembrane helices [4, 5]. When the primary structures of the two receptors from human are compared, 44% of the amino acid residues are identical over the entire length. In their transmembrane regions, the sequence identity increases to 68%. CB1 receptor exists in abundance at the presynaptic terminals in the various regions of the brain, including substantia nigra, striatum, hippocampus, and cerebral cortex, and negatively regulates the release of the neurotransmitters. CB1 is therefore the principal receptor mediating the psychoactivities of cannabis. CB1 receptor is also present in periphery such as adrenal gland, reproductive tissues, and immune cells at lower levels. On the other hand, CB2 receptor is mainly expressed in the immune system including the spleen, thymus, and lymph nodes and is involved in the immunomodulatory effects of cannabinoids. The expression levels of CB2 receptor in the human blood cells are in the following order: B cells > natural killer cells >> monocytes > polymorphonuclear neutrophil cells > CD8^+^ T cells > CD4^+^ T cells [9]. Activation of these receptors leads to a variety of cellular signal transduction such as a decrease in the cAMP level, an inhibition of N- and P/Q-type voltage-dependent Ca^2+^ channels, an opening of inwardly rectifying K^+^ channels, and an activation of mitogen-activated protein kinases.

Anandamide and 2-AG exert a variety of bioactivities as cannabinoid receptor ligands, including the cannabinoid tetrad: analgesia, catalepsy, hypolocomotion, and hypothermia. They also cause bradycardia and reductions of blood and intraocular pressures. As mentioned above, anandamide is a partial agonist of CB1 receptor, while 2-AG is a full agonist of both CB1 and CB2 receptors. Furthermore, the tissue levels of 2-AG are generally hundreds to thousands of times higher than those of anandamide. Thus, 2-AG is recognized to be the true endogenous ligands of CB1 and CB2 receptors and is considered to play more important roles in vivo than anandamide [10]. However, when the anandamide-degrading enzyme, fatty acid amid hydrolase (FAAH), is pharmacologically inhibited or genetically deficient, the local concentration of anandamide would rise and could exert CB1-dependent activities. It is important that 2-AG mediates retrograde signals at the synapse [11]. 2-AG is synthesized at the postsynaptic neurons in response to the stimulus of neurotransmitters such as glutamic acid. The released 2-AG then binds to and activates presynaptic CB1 receptors and inhibits the further release of the neurotransmitter.

In addition to CB1 and CB2 receptors, pharmacological studies suggest the presence of non-CB1, non-CB2 receptors mediating the effects of cannabinoids. Although several proteins have been discussed as candidates for such potential “CB3” receptor, its existence is controversial and not yet established [12]. One of the candidates is GPR55, a G protein-coupled receptor. Δ^9^-THC, a CB1/CB2 receptor agonist CP55940, anandamide, and 2-AG were reported to bind to GPR55 receptor overexpressed in human embryonic kidney HEK293s cells with nanomolar potencies, as analyzed with GTPγS binding experiments [13]. However, the pharmacological data of GPR55 gathered so far are conflicting and further analyses should be continued [14]. On the other hand, lysophosphatidylinositol, which is not a ligand of CB1 or CB2 receptor, was found to be the endogenous ligand of GPR55 [15]. Although this receptor can be activated by various molecular species of lysophosphatidylinositol having a different fatty acyl moiety at *sn*-1 or *sn*-2 position, 2-arachidonoyl-lysophosphatidylinositol is reported to be the most potent [16]. More recently, lysophosphatidylglucose was reported to be a more potent ligand of GPR55 and to mediate the correct guidance of nociceptive axons in the spinal cord [17]. Since anandamide also activates the transient receptor potential vanilloid type 1 (TRPV1) protein, a non-selective cation channel, anandamide is also regarded as one of endovanilloids [18]. However, its physiological significance as an endovanilloid is not fully elucidated.

## Biological activities of non-endocannabinoid *N*-acylethanolamines

Not only anandamide but also several ethanolamides of polyunsaturated fatty acids possessing three or more double bonds, such as dihomo-γ-linolenic acid (C20:3 ω6), mead acid (C20:3 ω9), and adrenic acid (C22:4), bind to cannabinoid receptors [19, 20]. However, saturated and monounsaturated *N*-acylethanolamines do not show ligand activity for cannabinoid receptors. Instead, these non-endocannabinoid *N*-acylethanolamines exert biological activities through different receptors. Importantly, non-endocannabinoid *N*-acylethanolamines such as palmitoylethanolamide (PEA, C16:0 *N*-acylethanolamine), stearoylethanolamide (C18:0 *N*-acylethanolamine), oleoyl-ethanolamide (OEA, C18:1 *N*-acylethanolamine), and linoleoylethanolamide (C18:2 *N*-acylethanolamine) are much more abundant than anandamide in most animal tissues. Biosynthetic enzymes for *N*-acylethanolamines so far reported do not show selectivity for anandamide over other *N*-acylethanolamine species. Thus, anandamide could be concomitantly produced as a kind of by-product of non-endocannabinoid *N*-acylethanolamines.

PEA is a food component known for more than 60 years [21]. This molecule was isolated from soybean lecithin, egg yolk, and peanut meal and was shown to exert an anti-inflammatory activity in a local passive joint anaphylaxis assay in the guinea pig [22, 23]. Since then, PEA has been shown to have anti-inflammatory, analgesic, anti-epileptic, and neuroprotective actions [24, 25]. These actions are mediated at least in part by PPARα. Preclinical and clinical studies suggest that PEA is potentially useful in a wide range of therapeutic areas, including eczema, pain, and neurodegeneration [26]. In the USA and Europe, PEA is currently marketed as a nutraceutical, a food supplement, or a food for medical purposes, depending on the country, which is effective for chronic pain represented by neuropathic pain. PEA is also a constituent of cream marketed for dry, irritated, and reactive skin. Although it was reported that PEA could activate GPR55 [13], this agonist activity has not been fully elucidated.

OEA is known to have an anorexic activity in experimental animals [27]. Administration of OEA produces satiety and reduces body weight gain [28]. OEA binds with high affinity to PPARα, and these effects are not observed with PPARα-deficient mice, suggesting that the anorexic action of OEA is mediated by PPARα. Since OEA is proposed to be produced from the digested dietary fat in the enterocytes of small intestine [29], endogenous OEA may mediate the satiety after the intake of fatty food. Furthermore, the dysfunction of OEA signaling could contribute to overweight and obesity. Thus, analogs of OEA and the inhibitors of OEA-degrading enzymes, such as FAAH, could be expected as novel anti-obesity drugs. OEA is also reported to activate GPR119 in vitro [30]. This G protein-coupled receptor was expressed in the intestinal L-cells, which secrete glucagon-like peptide-1 (GLP-1), and intraileal administration of OEA to rats was found to increase plasma GLP-1 levels [31]. However, the anorexic action of OEA was observed even in GPR119-deficient mice [32], suggesting that GPR119 system is not essential for OEA-induced satiety. Although OEA was reported to be a weak agonist of TRPV1 [33], TRPV1-deficient mice also exhibit OEA-induced suppression of appetite [34]. On the other hand, TRPV1 is suggested to mediate the reducing effects of OEA on levodopa (L-DOPA)-induced dyskinesia [35]. Thus, the OEA-TRPV1 system might be an effective target for the treatment of L-DOPA-induced dyskinesias.

Docosahexaenoylethanolamide (C22:6 *N*-acylethanol-amine) is the ethanolamide of docosahexaenoic acid, one of major ω3 polyunsaturated fatty acids, and is referred to as synaptamide. At nanomolar concentrations, synaptamide promotes neurogenesis, neurite outgrowth, and synaptogenesis in developing neurons [36]. Recently, these actions were shown to be mediated by the activation of GPR110, which is also termed as adhesion G protein-coupled receptor F1 (ADGRF1) [37]. Although the physiological significance in the development of neurons and cognitive functions remains elusive, the synaptamide-GPR110 system could be a novel target for the treatment of neurodevelopmental diseases. Furthermore, the beneficial effects of docosahexaenoic acid on the central nervous system might be partly mediated by the generation of synaptamide.

## Metabolism of endocannabinoid 2-arachidonoylglycerol

**Fig. 2:**
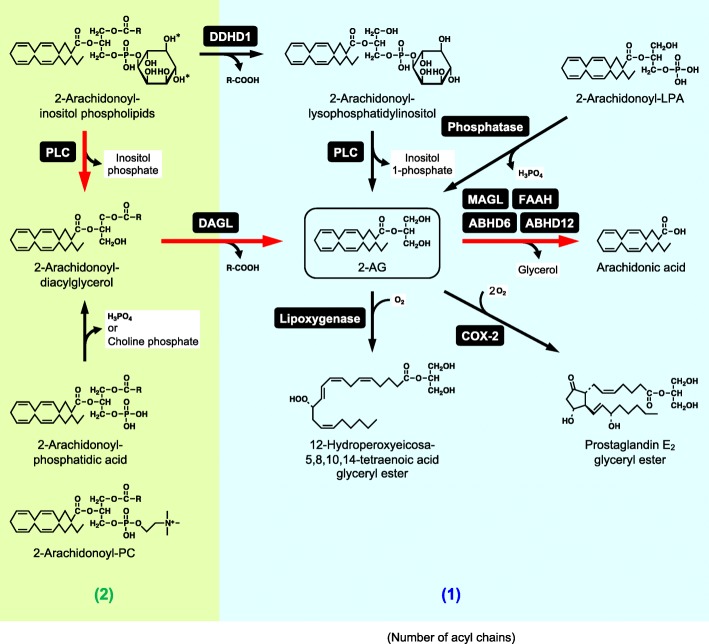
Metabolism of 2-AG. Red thick arrows represent the major pathway. H_2_O is omitted in the hydrolytic reactions. Two hydroxyl groups indicated by asterisks are phosphorylated in the case of 2-arachidonoyl-phosphatidylinositol 4,5-bisphosphate. Numbers of acyl chains per molecule are indicated in parentheses. *COX-2* cyclooxygenase-2, *DDHD1* DDHD domain containing 1, *PLC* phospholipase C

Although 2-AG is biosynthesized in multiple pathways, all the pathways start from *sn*-2 arachidonic acid-containing glycerophospholipids, which are abundant in cell membranes and therefore suitable as starting materials [10] (Fig. 2). The main precursors are inositol phospholipids with 2-arachidonoyl group such as 2-arachidonoyl-phosphatidylinositol 4,5-bisphosphate. The inositol phospholipids are hydrolyzed by phospholipase C to form 2-arachidonoyl-diacylglycerol, which is further deacylated by *sn*-1-specific diacylglycerol lipase (DAGL) to yield 2-AG (Fig. 2). Glycerophospholipids other than inositol phospholipids, such as phosphatidic acid and phosphatidylcholine (PC), could also be hydrolyzed to 2-arachidonoyl-diacylglycerol [38–40]. Human DAGL has two isozymes, DAGLα and DAGLβ. Their cDNAs were cloned in 2003 [41]. In DAGLα-deficient mice, the retrograde suppression of synaptic transmission is lost with concomitant decreases in 2-AG levels of brain and spinal cord [42–44]. Thus, DAGLα is suggested to be the main biosynthetic enzyme of 2-AG in the central nervous system. While the role of DAGL in the hydrolysis of membrane phospholipid-derived *sn*-1,2-diacylglycerol species is well established, it was described that DAGL enzymes are unlikely to be involved in the degradation of *rac*-1,3- or *sn*-2,3-diacylglycerol that originates from lipolysis-driven triacylglycerol breakdown [45].

Alternatively, 2-arachidonoyl-phosphatidylinositol could be hydrolyzed at *sn*-1 position by an intracellular phospholipase A_1_, DDHD domain containing 1, previously known as phosphatidic acid-preferring phospholipase A_1_ [46] (Fig. 2). The formed 2-arachidonoyl-lysophosphatidylinositol is known as an endogenous agonist of GPR55 as described above and is further hydrolyzed to 2-AG by a phospholipase C-type enzyme. Furthermore, 2-AG could be produced by dephosphorylation of arachidonic acid-containing lysophosphatidic acid (LPA) [47]. These alternative pathways, which bypass 2-arachidonoyl-diacylglycerol and therefore do not involve DAGL, seemed to play a certain role in vivo since ~ 15% of 2-AG levels remained even in the cerebral cortex of DAGLα/β double-knockout mice, compared to those of wild-type mice [44].

The major degradative pathway of 2-AG is considered to be the hydrolysis to arachidonic acid and glycerol (Fig. 2). This reaction can be catalyzed by multiple enzymes, including monoacylglycerol lipase (MAGL), FAAH, α/β-hydrolase domain containing (ABHD) 6, and ABHD12. The relative contribution of these enzymes differs among tissues and cells. In mouse brain, MAGL is responsible for around 85% of the 2-AG-hydrolyzing activity in vitro [48]. cDNA of this enzyme was cloned from mouse adipocytes in 1997 [49]. MAGL hydrolyzes not only 2-AG but also other 2-monoacylglycerols and 1-monoacylglycerols. Pharmacological inhibition of MAGL in mice caused CB1-dependent symptoms including analgesia, hypothermia, and hypomotility, indicating the central role of this enzyme in the degradation of 2-AG in the brain [50]. Although MAGL-deficient mice exhibited increased 2-AG levels in the brain and spinal cord, no abnormalities in nociception, body temperature, or spontaneous locomotion were observed in MAGL-deficient mice [51, 52]. This apparent discrepancy is supposed to be due to the desensitization of CB1 receptor. Apart from the endocannabinoid system, MAGL-dependent generation of arachidonic acid from 2-AG is also responsible for the production of prostaglandins that promote neuroinflammation and fever generation in the brain [53, 54].

FAAH plays the central role in the degradation of anandamide, another endocannabinoid, as described in the following section. FAAH also hydrolyzes 2-AG. However, the role of FAAH in 2-AG degradation in vivo is considered to be minor. In mouse microglia BV-2 cells, ABHD6 controls the accumulation of 2-AG, and knockdown of ABHD6 increases the efficacy with which 2-AG can stimulate CB2-mediated cell migration [55]. ABHD6 is also expressed postsynaptically in neurons, and the specific inhibitor of ABHD6 as well as MAGL inhibitors induces CB1-dependent long-term depression. As another metabolic route of 2-AG, the arachidonoyl moiety of 2-AG could be directly oxygenated by cyclooxygenase-2 and lipoxygenases to produce glycerol esters of prostaglandins and hydroperoxyeicosatetraenoic acids, respectively (Fig. 2). Glycerol esters of prostaglandins are reported to show biological activities including anti-inflammatory, pro-inflammatory, and hyperalgesic effects [56].

The pathway consisting of phospholipase C, DAGL, and MAGL has attracted attention due to the formation of two second messengers, diacylglycerol and inositol trisphosphate, and the release of free arachidonic acid from phospholipid, which may be utilized to generate eicosanoids. The major pathway for the biosynthesis and degradation of 2-AG completely agrees with this pathway, and this fact implies its multifunctionality of this pathway.

## Metabolism of *N*-acylethanolamines

**Fig. 3:**
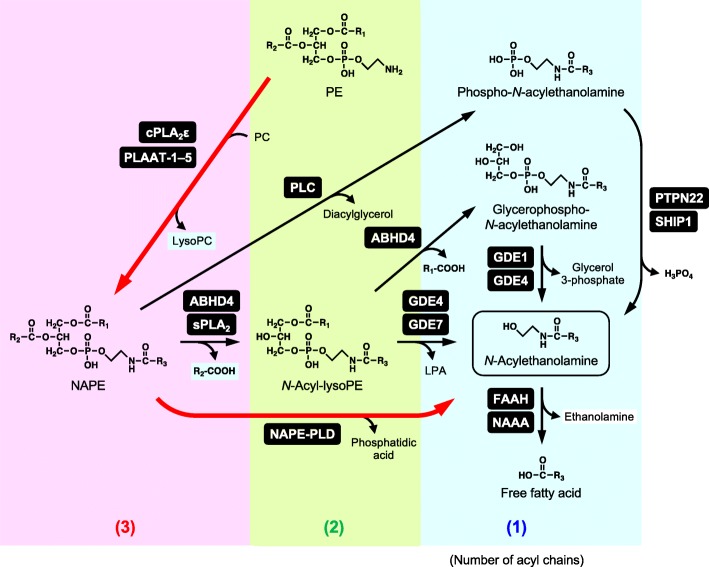
Metabolism of *N*-acylethanolamines. Red thick arrows represent the canonical pathway. H_2_O is omitted in the hydrolytic reactions. Numbers of acyl chains per molecule are indicated in parentheses. *cPLA*_*2*_ cytosolic phospholipase A_2_, *PLC* phospholipase C, *sPLA*_*2*_ secretory phospholipase A_2_

In animal tissues, a series of *N*-acylethanolamines including anandamide is biosynthesized through common metabolic pathways starting from glycerophospholipids (Fig. 3). The pathways are largely different from the aforementioned 2-AG metabolism. First, *sn*-1 acyl group of glycerophospholipids such as PC is transferred to the amino group of ethanolamine glycerophospholipids represented by phosphatidylethanolamine (PE). This *N*-acylation of PE results in the generation of *N*-acyl-PE (NAPE), which is a unique type of glycerophospholipid in that three fatty acyl chains exist per molecule. The responsible enzyme *N*-acyltransferase has been known to be stimulated by Ca^2+^ since the 1980s [57–59] and called as Ca-dependent *N*-acyltransferase (Ca-NAT) to distinguish from Ca-independent enzymes discussed later. However, its molecular characterization was achieved only recently when mouse Ca-NAT was identified by an activity-based proteomic approach as isoform ε of cytosolic phospholipase A_2_ (PLA2G4E) [60]. Our group then found that human ortholog has two isoforms, which are distinguished by the length and amino acid residues of their N-terminal sequences, and that both isoforms show Ca-NAT activity [61]. We also revealed that this Ca^2+^-dependent activity is further enhanced by phosphatidylserine. In agreement with the fact that the *sn*-1 position of glycerophospholipids is mostly occupied by a saturated or monounsaturated fatty acid, the anandamide precursor *N*-arachidonoyl-PE is a minor component among various NAPEs with different *N*-acyl species. This may be the main reason why anandamide is a minor component of *N*-acylethanolamines.

Apart from Ca-NAT, we found that all of the five members of HRAS-like suppressor (HRASLS) family, HRASLS1–5, have Ca^2+^-independent *N*-acyltransferase activities as well as phospholipase A_1_/A_2_ activities [62–67]. These family members were previously reported as tumor suppressor genes, negatively regulating the oncogene *Ras*. On the basis of their enzyme activities, we proposed to rename them phospholipase A/acyltransferase (PLAAT)-1–5, respectively [66]. Among the members, PLAAT-1, PLAAT-2, and PLAAT-5 have relatively high *N*-acyltransferase activities over phospholipase A_1_/A_2_ activities [67, 68], suggesting their roles in the Ca^2+^-independent generation of NAPE in vivo.

The formed NAPE is then hydrolyzed to release *N*-acylethanolamines by a phospholipase D (PLD)-type enzyme, NAPE-PLD (Fig. 3). Our group purified this enzyme from rat heart and cloned its cDNAs from human, mouse, and rat [69]. The enzyme specifically hydrolyzes NAPE, but not PE or PC. The primary structure of NAPE-PLD shows that this enzyme belongs to the metallo-β-lactamase family and has no sequence similarity with other PLDs, which typically hydrolyze PC to phosphatidic acid and choline. Thus, NAPE-PLD is distinct from other PLDs in both structure and catalytic function.

In addition to the one-step *N*-acylethanolamine-forming reaction catalyzed by NAPE-PLD, the presence of multi-step pathways via *N*-acyl-lysoPE was suggested using dog brain preparations in the 1980s [58] (Fig. 3). The cDNA cloning of NAPE-PLD enabled the generation of NAPE-PLD^−/−^ mice, and three groups including ours independently established the mutant mice and confirmed the presence of the multi-step NAPE-PLD-independent pathways in brain and other mammalian tissues [70–73]. In these pathways, one *O*-acyl chain is first eliminated from NAPE, resulting in the formation of *N*-acyl-lysoPE. This reaction occurred in vitro by group IB, IIA, and V of secretory phospholipase A_2_s [74]. *N*-Acyl-lysoPE can be further *O*-deacylated to glycerophospho-*N*-acylethanol-amine. ABHD4 was found to function as a hydrolase catalyzing these sequential *O*-deacylation reactions from NAPE to glycerophospho-*N*-acylethanolamine via *N*-acyl-lysoPE [75]. Glycerophospho-*N*-acylethanolamine is further hydrolyzed to form *N*-acylethanolamine by two members of the glycerophosphodiesterase (GDE) family, GDE1 [76] and GDE4 [77, 78]. Alternatively, *N*-acyl-lysoPE can be directly converted to *N*-acyletha-nolamine by lysophospholipase D-type enzymes. In this reaction, LPA is also formed as another product. This lysophospholipase D-type reaction seems particularly important when the substrate *N*-acyl-lysoPE is “plasmalogen-type” containing a lipase-resistant alkenyl chain at *sn*-1 position of the glycerol backbone [71]. We found that GDE4 and GDE7 have this lysophospholipase D-type activity [77, 78]. Interestingly, the divalent cation requirement for the activity differs among GDE members: GDE1 and GDE4 are Mg^2+^-dependent while GDE7 is Ca^2+^-dependent. In addition, an anandamide-forming pathway through phosphoanandamide (anandamide phosphate) was previously suggested in the brain and macrophages. This pathway is composed of phospholipase C and phosphatase. Tyrosine phosphatase PTPN22 and inositol 5′-phosphatase SHIP1 were shown to have this phosphatase activity while the phospholipase C has not yet been identified [79, 80]. The reverse reaction of FAAH can synthesize anandamide from free arachidonic acid and ethanolamine in vitro [81, 82]. The analysis of FAAH-deficient mice suggests the in vivo production of anandamide through this route [83].

*N*-Acylethanolamines are degraded by the hydrolysis to free fatty acids and ethanolamine (Fig. 3). FAAH catalyzes this reaction, and this enzyme has been extensively studied since its cDNA cloning in 1996 [84]. FAAH is a membrane-bound serine hydrolase, belonging to the amidase signature family. The catalytic activity is higher at neutral and alkaline pH. FAAH hydrolyzes various *N*-acylethanolamines with a higher reactivity toward anandamide. FAAH is ubiquitously present in various tissues with abundant expressions in the brain and liver, and FAAH-deficient mice exhibit increased tissue levels of various *N*-acylethanolamines including anandamide, suggesting the central role of this enzyme in the degradation of *N*-acylethanolamines [85, 86]. Specific FAAH inhibitors have been developed, and they are expected as novel therapeutic drugs against a variety of symptoms such as pain, depression, and anxiety. These beneficial effects are mostly considered to result from the increased tissue levels of anandamide acting as an endocannabinoid. However, FAAH also hydrolyzes cannabinoid receptor-insensitive *N*-acylethanolamines and other bioactive fatty acid amides such as oleamide and *N*-acyltaurine. Thus, we should be careful in interpreting the molecular mechanisms of the phenotype caused by genetic and pharmacological depletion of FAAH. The dual inhibitors of FAAH and MAGL have also been developed, and they increase both anandamide and 2-AG levels to mimic the pharmacological activities of CB1 receptor agonist in vivo [87, 88]. FAAH-2, an isozyme having around 20% of amino acid sequence identity with FAAH (FAAH-1), is also present in primates, but not in rodents [89], and this enzyme localizes on lipid droplets in cells [90].

*N*-Acylethanolamine-hydrolyzing acid amidase (NAAA) is a lysosomal enzyme hydrolyzing *N*-acylethanolamines only at acidic pH [91]. We cloned cDNA of this enzyme from rat lung in 2005 [92]. NAAA belongs to the cholylglycine hydrolase family and shows no sequence similarity with FAAH. Acid ceramidase is another lysosomal enzyme belonging to this family, which hydrolyzes ceramide under acidic conditions. NAAA and acid ceramidase have significant amino acid sequence similarity (33–34% identity), and their catalytic activities partially overlap each other: NAAA hydrolyzes ceramide at a low rate while acid ceramidase also has an *N*-acylethanolamine-hydrolyzing activity. NAAA is present in various tissues with abundant expression in macrophages and prostate [93, 94]. In contrast to the preference of FAAH to anandamide, the best substrate of NAAA in vitro is PEA. In consistence with the anti-inflammatory action of PEA, the administration of specific NAAA inhibitors suppresses inflammatory responses in rodent models with increased local PEA levels [95–99]. NAAA-deficient mice also show a strongly reduced inflammatory reaction, compared to wild-type animals [99]. Thus, NAAA inhibitors may have the therapeutic potential as novel anti-inflammatory drugs.

## Conclusions

In this mini-review, we outlined the biological activities and metabolisms of two representative endocannabinoids, 2-AG and anandamide, as well as cannabinoid receptor-insensitive *N*-acylethanolamines. Pharmacological and biochemical analyses now reveal that 2-AG is a more important endocannabinoid than anandamide. The classical pathway composed of phospholipase C, DAGL, and MAGL attracts much attention again as the central pathway for the metabolism of 2-AG functioning as the major endocannabinoid. On the other hand, anandamide is produced in a small amount along with PEA and OEA, which are cannabinoid receptor-insensitive, but quantitatively major bioactive *N*-acylethanolamines. The presence of Ca-NAT and NAPE-PLD, which appear to be exclusively responsible for the biosynthesis of *N*-acylethanolamines, strongly suggest the physiological importance of *N*-acylethanolamines and their precursors *N*-acyl-PEs. Thus, further studies on biological activities of various *N*-acylethanolamines are eagerly required, which include the development of specific enzyme inhibitors and analyses of gene-disrupted animals for the enzymes involved. As the research in this field progresses, the metabolic pathways have been found to be more complex than previously considered. Recently found enzymes, such as PLAAT and GDE family members, have not been fully elucidated and their roles in vivo must be clarified.

## Abbreviations

2-AG: 2-Arachidonoylglycerol
ABHD: α/β-Hydrolase domain containing
Ca-NAT: Ca-dependent *N*-acyltransferase
DAGL: Diacylglycerol lipase
FAAH: Fatty acid amide hydrolase
GDE: Glycerophosphodiesterase
GLP-1: Glucagon-like peptide-1
HRASLS: HRAS-like suppressor
LPA: Lysophosphatidic acid
MAGL: Monoacylglycerol lipase
NAAA: *N*-Acylethanolamine-hydrolyzing acid amidase
NAPE: *N*-Acyl-phosphatidylethanolamine
OEA: Oleoylethanolamide
PC: Phosphatidylcholine
PE: Phosphatidylethanolamine
PEA: Palmitoylethanolamide
PLAAT: Phospholipase A/acyltransferase
PLD: Phospholipase D
PPARα: Peroxisome proliferator-activated receptor α
TRPV1: Transient receptor potential vanilloid type 1
